# Under-Capture of Candidates Supported by Temporary Mechanical Circulatory Support in the OPTN STAR Registry

**DOI:** 10.1016/j.jacadv.2026.103213

**Published:** 2026-09-10

**Authors:** Daniel J. Ahn, Antony Attia, Toshihiro Nakayama, William F. Parker, Kiran K. Khush

**Affiliations:** a Stanford University, Stanford, California, USA; b Stanford Transplant Outcomes Research Center, Stanford University, Stanford, California, USA; c Stanford Cardiovascular Institute, Stanford University, Stanford, California, USA; d University of Chicago, Chicago, Illinois, USA

We read with interest the study by Cyrille-Superville et al^1^ examining sex-specific trends in temporary mechanical circulatory support (MCS) use as a bridge to heart transplantation using the Standard Transplant Analysis and Research (STAR) registry from the Organ Procurement and Transplantation Network (OPTN). We commend the authors for this important contribution but wish to raise a methodological concern regarding the data source.

Heart transplant programs submit MCS device information to the OPTN through multiple instruments. Between initial listing and waitlist removal, programs report MCS use through status justification forms to document ongoing device support. They also submit MCS removal forms when candidates are taken off of the waitlist. The supplemental MCS file in the STAR registry is constructed exclusively from the removal forms, capturing device status only for candidates who have exited the waitlist. As a result, candidates who are still actively listed, even with ongoing device support, are systematically excluded.

We recently demonstrated the magnitude of this bias for durable left ventricular assist devices (LVADs),^2^ showing an artifactual decline in LVAD prevalence among waitlisted candidates from 34.8% in October 2018 to 3.2% in April 2025 when relying on the STAR MCS file alone, vs stable prevalence (30.7% to 37.4%) when supplementing the MCS file with status justification forms. Importantly, this systematic undercounting applies to all MCS devices, not just durable LVADs.

**Figure 1:**
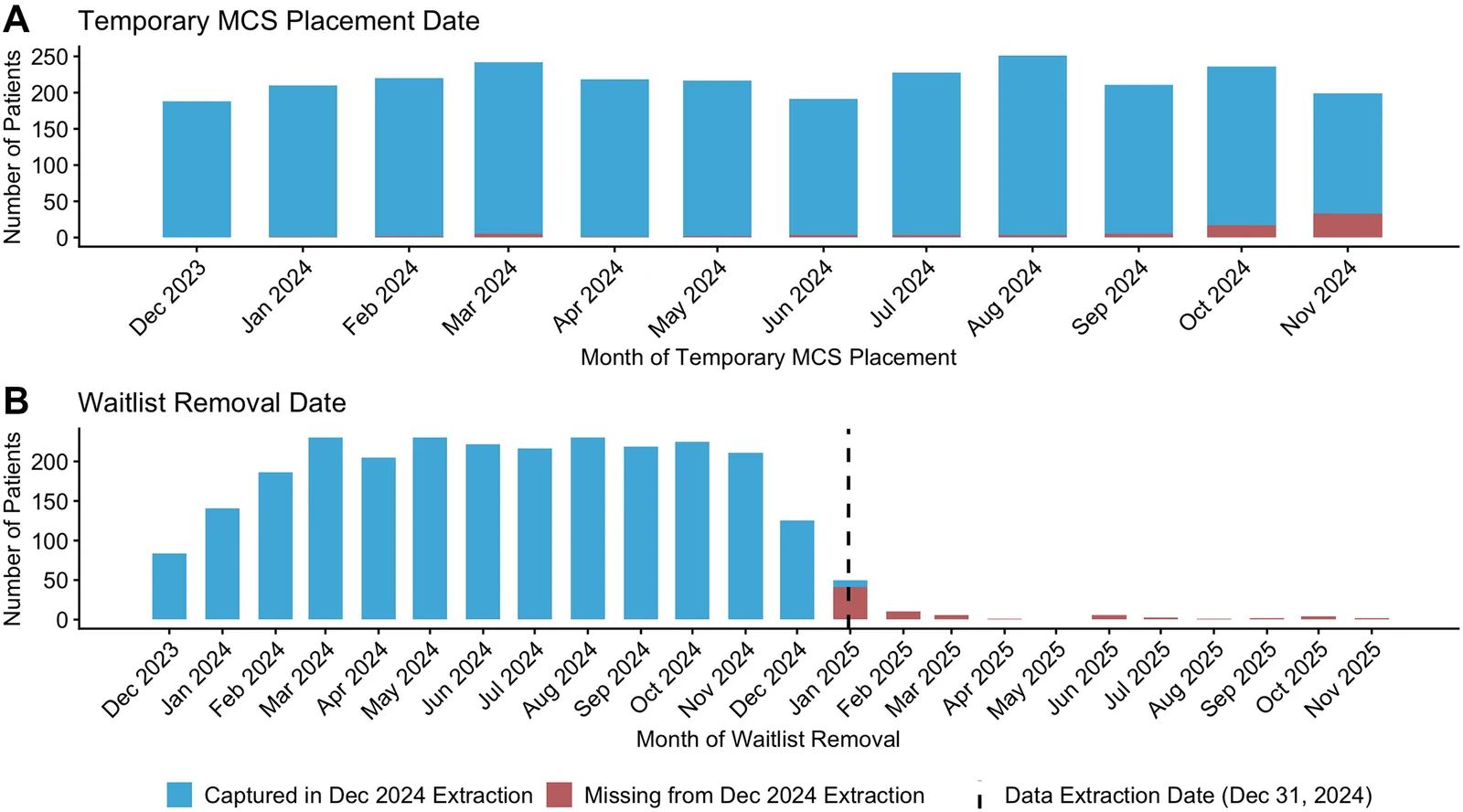
Retroactive Ascertainment of Temporary MCS-Supported Adult Candidates (A) Shows the month of temporary MCS placement for all 2,611 adult candidates identified using a December 31, 2025, data extraction with temporary MCS (IABP, nondischargeable or percutaneous LVAD, TAH, VA ECMO) placed between December 1, 2023, and November 30, 2024. (B) Shows the month of waitlist removal for the same cohort. Patients captured in the December 31, 2024, extraction (N = 2,533) are shown in blue. Patients missing from the December 2024 extraction (N = 78) are shown in red. All 78 missing patients had active temporary MCS support prior to December 31, 2024, but appeared in the STAR MCS file only after being removed from the waitlist in 2025. The dashed vertical line denotes the data extraction date (December 31, 2024). MCS = mechanical circulatory support.

To illustrate this, we identified all adult candidates with temporary MCS (using the same definition as in Cyrille-Superville et al^1^) placed between December 1, 2023, and November 30, 2024, using a December 31, 2024, data extraction from the OPTN STAR registry, identifying 2,533 candidates. These data were obtained under a data use agreement with the OPTN under exemption by the Stanford University Institutional Review Board. When we repeated the identical query with a December 31, 2025, data extraction, the cohort expanded to 2,611, an increase of 78 patients (3.0%). These 78 patients were supported by temporary MCS as early as January 2024. However, they were absent from the December 31, 2024, extraction because they were still actively listed by December 31, 2024. They only appeared after removal from the waitlist through December 31, 2025, at which point their device records were retroactively captured (Figure 1). This confirms that even in a population with relatively short median waitlist times, the STAR MCS file’s structural exclusion of currently listed candidates produces a measurable, time-dependent undercount.

The present study^1^ does not report the date of STAR file extraction relative to cohort closure (June 28, 2023) or which data files were used to identify the MCS devices. This omission is relevant because any temporary MCS-supported candidates still listed at the time of extraction would be excluded if the authors only utilized the MCS file. Given that inclusion in the MCS file requires a waitlist outcome, such exclusion would systematically underestimate true temporary MCS prevalence and could disproportionately affect one sex over the other if listing duration or removal timing differs by sex, potentially skewing the sex-stratified comparisons central to the study’s conclusions.
